# Photon irradiation using a water bath technique for treatment of confluent carcinoma *in situ* of the hand, digits, and nail bed: a case report

**DOI:** 10.1186/s13256-017-1233-3

**Published:** 2017-03-30

**Authors:** Chelain R. Goodman, Albert DeNittis

**Affiliations:** 1 Department of Internal Medicine, Lankenau Medical Center, 100 Lancaster Avenue, Wynnewood, PA 19096 USA; 2 Department of Radiation Oncology, Lankenau Medical Center, 100 Lancaster Avenue, Wynnewood, PA 19096 USA

**Keywords:** Radiation, Carcinoma *in situ*, Bowen’s disease, Squamous cell carcinoma, Digits, Nail bed, Case report

## Abstract

**Background:**

Confluent squamous cell carcinoma *in situ,* or Bowen’s disease, involving the hand, digit, and nail bed is rare and represents a significant therapeutic challenge. Surgical excision is recommended as first-line treatment but in cases of extensive disease can lead to unacceptable functional morbidity or cosmetic outcomes. Radiation therapy has been shown to be equally efficacious to surgery in the treatment of carcinoma *in situ* but its use has historically been limited due to concerns regarding toxicity. In this case report we present a novel therapeutic technique that may enable radiotherapy to be employed as a definitive treatment option in these challenging cases.

**Case presentation:**

A 75-year-old white man with a previous history of carcinoma *in situ* of his right hand previously treated with 5-fluorouracil presented with recurrent biopsy-proven confluent squamous cell carcinoma *in situ* of multiple surfaces of his right hand and digits with involvement of nail beds. To avoid extensive resection and possible amputation he was offered definitive external beam radiation therapy utilizing a water bath as a tissue-equivalent bolus material. This protocol enabled improved dose homogeneity to the target volume while minimizing acute toxicity. He experienced complete clinical resolution of the disease with only minimal acute edema and hyperpigmentation. Twenty months following treatment completion he remains disease-free with normal function and excellent cosmesis.

**Conclusions:**

Therapeutic radiation utilizing water as a tissue-equivalent bolus in this complicated case enabled definitive treatment of disease without compromising functional or cosmetic outcomes. Radiotherapy may therefore be an alternative and under-utilized approach to surgical excision in difficult-to-treat cases of carcinoma *in situ*.

## Background

Skin cancer is the most commonly diagnosed cancer in the USA; over three million patients are diagnosed as having basal cell or squamous cell carcinoma (SCC) each year [1]. Squamous cell carcinoma *in situ* (CIS), or Bowen’s disease, refers to the pre-invasive stage of the disease in which the cancer is confined to the epidermal layer. If left untreated, 3 to 5% of these *in situ* lesions will ultimately progress to invasive SCC and one-third of these invasive cancers may metastasize [1].

Confluent and extensive CIS, particularly involving digits and nail bed, represents a rare yet significant therapeutic challenge. Surgical excision is recommended as first-line treatment but in cases of extensive disease can lead to unacceptable functional morbidity or cosmetic outcomes [1]. External beam radiation therapy (EBRT) utilizing photon or electron beams has been demonstrated to lead to equivalent recurrence rates when compared to surgical excision in cases of CIS of the hand and nail bed [2–5]. Its use, however, has been limited due to concerns regarding toxicity [6]. Lesions of the digit and nail bed are particularly challenging in radiation treatment planning as the irregular surfaces of these sites obligate customized bolusing to achieve homogenous surface dosing [6].

Here we discuss a rare case of extensive CIS involving multiple surfaces of the hand with extension to several digits and nail beds. Definitive EBRT employing a water bath technique is presented as an effective modality in treating extensive CIS with excellent clinical, functional, and cosmetic outcomes.

## Case presentation

A 75-year-old white man with a previous history of CIS of his right hand treated with topical 5-fluorouracil (5-FU) presented with new extensive lesions of his right hand. A dermatological examination demonstrated a confluent erythematous plaque covering the ventral, lateral, and dorsal surfaces of his right hand involving the digits and the second through to fourth nail beds. Shave biopsies revealed atypical keratinocytes throughout the epidermal layer consistent with CIS.

**Fig. 1:**
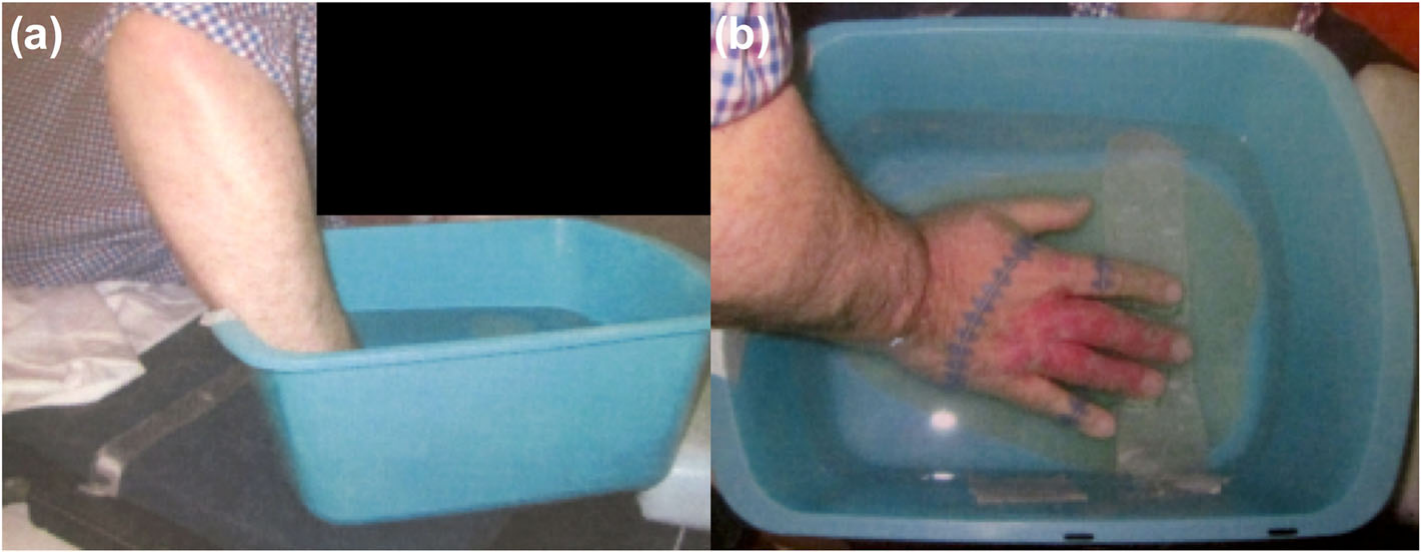
Side (**a**) and top-down (**b**) views of the clinical set-up demonstrating the water basin filled to 2 cm above the dorsal surface of the extremity as well as the 1 cm Superflab bolus below the ventral surface

**Fig. 2:**
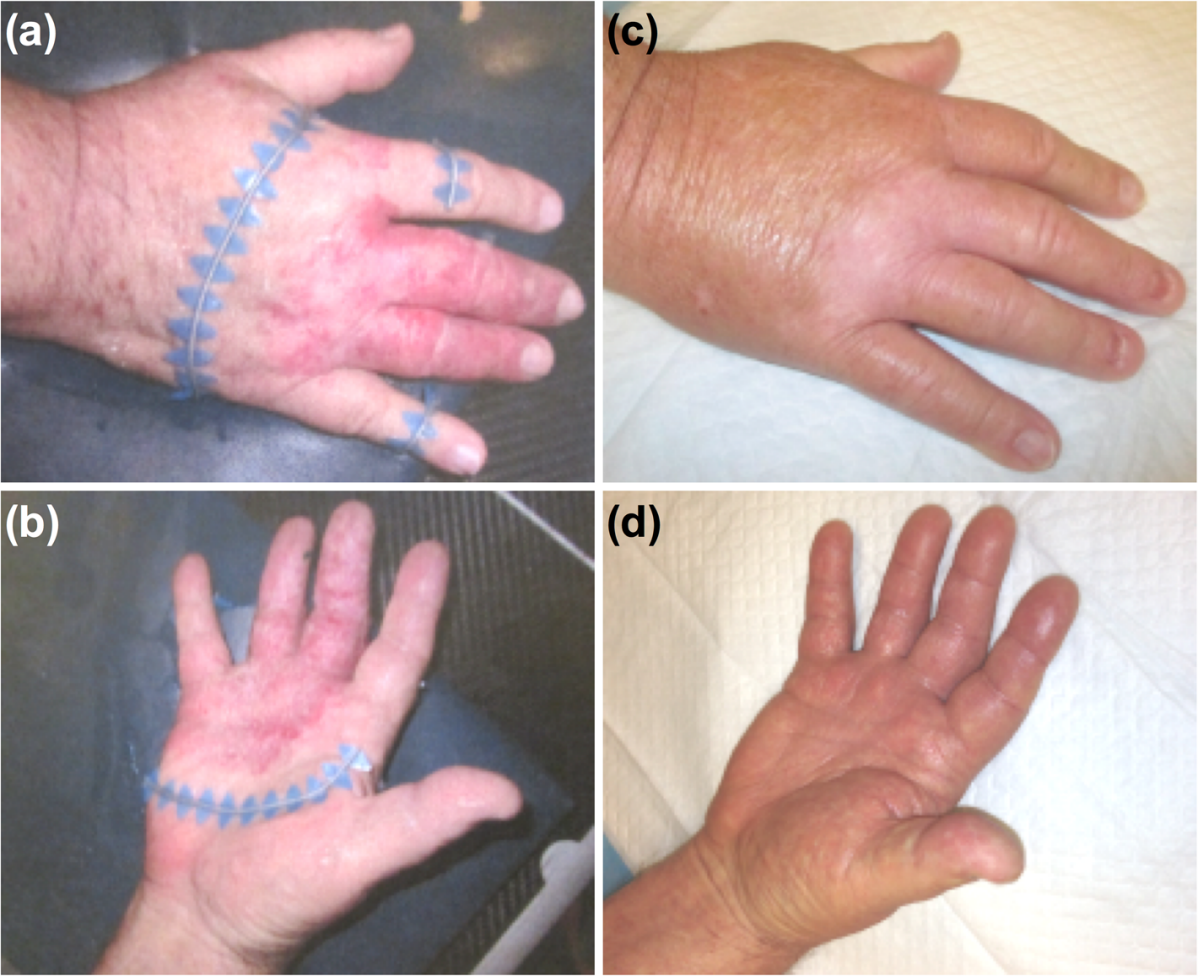
Dorsal (**a**) and ventral (**b**) surfaces of the right hand prior to treatment during simulation. Dorsal (**c**) and ventral (**d**) surfaces of the right hand 3 months following treatment completion

Recommended surgical options would have probably required extensive skin grafting or amputation. He was therefore offered definitive EBRT utilizing a water bath as a tissue-equivalent bolus. He was simulated via computed tomography (CT) with all visible disease delineated with wire. His hand was immersed in a water basin filled to 2 cm above the dorsal surface, with 1 cm of tissue-equivalent Superflab below the ventral surface (Fig. 1a, b). His first and fifth digit nail beds were blocked using multileaf collimation. Sixty Gray (Gy) over 30 fractions were delivered using 6 megavoltage (MV) photons normalized to the 95% isodose line. Acute side effects were notable for only mild edema and hyperpigmentation. Three months following completion of treatment there was complete clinical resolution of disease (Fig. 2a-d). He exhibited excellent skin healing and remained without loss-of-function or neuropathy. At 20 months following treatment completion, he is without evidence of disease recurrence or complication.

## Discussion and conclusions

CIS can progress to invasive SCC and requires aggressive treatment. Most commonly seen on skin regions chronically exposed to ultraviolet light radiation such as the head and neck, CIS has also been described of the vulva, anus, palm, digit, and nail unit [7]. CIS typically presents as a slowly-enlarging, well-demarcated erythematous plaque with a scaling surface and can range in size, with disease greater than 3 cm being less common [1].

A variety of treatment options are available for CIS, including surgical excision, Mohs surgery, cryotherapy, photodynamic therapy (PDT), and therapeutic radiation [1]. While surgical excision is recommended as first-line treatment for CIS, EBRT has been shown to be similarly efficacious to surgical excision and is recommended in cases of extensive disease or for surgically ineligible patients [8]. Retrospective studies evaluating EBRT in the treatment of CIS have demonstrated local control rates between 93 and 100% [2, 9]. Widespread use of therapeutic radiation in the treatment of CIS, however, has been historically limited due to reports of poor healing, particularly for lesions on irregular surfaces [6].

Confluent disease greater than 3 cm is rare and presents a particular challenge in achieving disease control while also providing acceptable cosmetic and functional outcomes [1]. Several case reports have described innovative radiotherapeutic techniques with excellent local recurrence and cosmetic results. A high-energy beta-emitter holmium 166 skin patch [10] as well as high-dose rate (HDR) brachytherapy utilizing iridium-192 [11] have both been utilized to treat extensive CIS with complete resolution and no sign of recurrence at 14 to 24 months following treatment. Similarly, combination therapy of PDT followed by EBRT with electrons demonstrated excellent crude control without recurrence [12].

Mohs surgery has been considered the standard of care for large lesions of the hand and digits, but can lead to contractures, loss of the fingernail, or amputation [13]. Until recently, EBRT was rarely utilized in these cases due to high reported recurrence rates and difficulties achieving consistent surface dosing [14]. More recent case reports utilizing electron beams [5], photon beams [4], as well as HDR brachytherapy [11] in the treatment of CIS and SCC of the nail and digit have reported excellent functional outcomes without evidence of recurrence at 1 to 4 years following treatment. Similar to our protocol, Herman *et al.* [3] utilized a water bath as a bolus for photon irradiation in nine patients with small lesions of the digit. All but one patient in this series recovered normal function and no recurrences were noted, although several patients were evaluated at short follow-up points.

The case presented in this report is unusual due to the extent and confluence of the disease encompassing multiple surfaces of the hand and digits, the involvement of multiple nail beds, as well as its recurrent status refractory to topical 5-FU. We utilized a water bath with Superflab to function as a tissue-equivalent bolus to negate the skin-sparing properties of megavoltage photon irradiation. This technique enabled the production of a uniform surface dose for optimal target volume dosing on all surfaces while minimizing radiation toxicity [15]. The treatment regimen was well-tolerated with only minimal acute toxicity. At 3 months the patient experienced complete disease resolution. He remains free of evidence of recurrence more than 20 months from treatment completion and displays normal functional use and excellent cosmesis. Therapeutic radiation utilizing a water bath may therefore be an under-utilized treatment modality for CIS, particularly for patients at high risk of functional morbidity following surgical excision. Clinical studies that further examine the efficacy and toxicity of radiotherapy in the treatment of large confluent cases of CIS not amenable to surgical resection may be warranted.

## Abbreviations

5-FU: 5-fluorouracil
CIS: Carcinoma *in situ*
CT: Computed tomography
EBRT: External beam radiation therapy
Gy: Gray
HDR: High-dose rate
MV: Megavoltage
PDT: Photodynamic therapy
SCC: Squamous cell carcinoma
